# Root-derived AMF communities modulate growth and nutrient dynamics in grapevine rootstocks

**DOI:** 10.1007/s00572-026-01264-5

**Published:** 2026-05-07

**Authors:** Romy Moukarzel, Hayley J. Ridgway, Lauren Waller, Alexis Guerin-Laguette, Natalia Cripps-Guazzone, E. Eirian Jones

**Affiliations:** 1 Department of Pest-management and Conservation, Lincoln University, Lincoln, 7647 New Zealand; 2 Canterbury Agriculture & Science Centre, The New Zealand Institute for Bioeconomy Science Limited, Gerald St, Lincoln, 7608 New Zealand; 3 Mycotree C/-Southern Woods Nursery, 1002 Robinsons Rd, RD8, Christchurch, 7678 New Zealand

**Keywords:** *Vitis vinifera*, Rootstocks, Spores, Arbuscular mycorrhizal fungi, Symbiosis

## Abstract

Arbuscular mycorrhizal fungi (AMF) play vital roles in sustainable agriculture, yet evidence linking AMF community composition to plant benefits remains limited. To address this gap, we inoculated two commercial rootstocks (Schwarzmann and 5 C) with AMF communities recovered from different rootstocks from one site to determine effects on plant growth parameters and physiological responses. A glasshouse experiment using a ‘home’ and ‘away’ approach was designed to examine the interaction between rootstock variety and different AMF communities, including those from their own (‘home’) and other rootstocks’ rhizosphere soils (‘away’). Our results showed that rootstocks grown in their ‘home’ AMF communities exhibited greater above and below ground biomass compared to ‘away’ AMF communities, highlighting rootstock specificity in selecting AMF communities. AMF communities increased chlorophyll content and nutrient uptake (copper, boron) in grapevine leaves, where AMF communities dominated by *Funneliformis* sp., *Ambispora* sp. followed by *Glomus* spp. were associated with enhanced grapevine growth. This study enhances our understanding of community-level AMF-grapevine interactions and highlight the ecosystem services these fungi provide. Future research is needed using grafted plants to evaluate their response with different scions following AMF inoculation and to assess the effects of these AMF communities on berry biochemical composition.

**Supplementary Information:**

The online version contains supplementary material available at 10.1007/s00572-026-01264-5.

## Introduction

Arbuscular mycorrhizal fungi (AMF) are obligate biotrophs that can form a symbiotic interaction with more than 80% of all land plant families by colonizing their roots (Smith and Read 2008). AMF depend on plant photosynthetic products (Kiers et al. 2011; Bonfante and Genre 2010) and lipids to complete their life cycle (Xu et al. 2017). These fungi can influence plant diversity and ecosystem productivity (Heijden et al. 2015). AMF have a considerable carbon cost for the host plant (Begum et al. 2019), receiving four to twenty% of the plant’s photosynthetically fixed carbon (Smith and Read 2008). Consequently, when the carbon cost of maintaining the mycorrhizal symbiosis exceeds the nutritional benefits to the host, plant growth can be negatively affected (Smith et al. 2011). This reflects the broader concept that mycorrhizal interactions operate along a continuum from mutualism to parasitism, depending on environmental conditions, host status, and fungal identity (Martin et al., 2025).

In viticulture, the AMF-grapevine interaction is considered as an important element of the vineyard system (*see reviews* by Trouvelot et al. 2015; Velaz et al. 2025). Inoculating grapevines with AMF leads to enhanced growth rate and nutrient uptake as well as drought tolerance compared to non-inoculated grapevines (Karagiannidis et al. 2007; Nogales et al. 2019; Torres et al. 2021; Valenzuela-Aragon et al. 2025). It is reported that grapevine rootstock cuttings inoculated with each of two *F. mosseae* isolates that had been recovered from two different soils revealed that only the isolate native to the high P soil enhanced Cu and S uptake (Schreiner 2007). A recent study identified distinct AMF communities associated with different grapevine rootstocks in one vineyard site (Moukarzel et al. 2021). Interestingly, in the rootstocks with similar parentage did not cluster in the AMF communities and seemed to select the AMF communities which were beneficial to their development (Moukarzel et al. 2022). Recent work has also demonstrated that both grapevine variety and rootstock can significantly influence the composition of fungal and arbuscular mycorrhizal communities associated with vines, even within a single vineyard site (Noceto et al. 2024).

Single-species inoculations with various AMF species have shown that grapevines rely on AMF for increases in root and shoot biomass (Menge et al. 1983; Karagiannidis et al. 2007), elongation (Gloire, St. George, 3309 C) and photosynthetic rates (Mortimer et al. 2004), but the benefits derived from AMF appear to vary with the rootstock and AMF species combination (Lindermann and Davis, 2001; Bleach et al. 2008; Nogales et al. 2009; Cangahuala-Inocente et al. 2011). In another study, it was shown that grapevine response to AMF inoculation with *Rhizophagus intraradices* (as *G. intraradices)* was influenced by the intrinsic characteristics of the vineyard soil, the rootstock used and the time after planting (Nogales et al. 2009). A recent study also demonstrated that rootstock genotype and type of AMF inoculum could influence the potential benefits of mycorrhizal inoculation and thus the response of young vines to the environment (Holland et al. 2018). The knowledge gap in global AMF-grapevine interactions hinders the potential applications of arbuscular mycorrhizal fungi (AMF) in sustainable agriculture. Native AMF species show greater effectiveness than non-native species when associated with *Vitis vinifera* (Schreiner et al. 2007; Massa et al. 2020). This means that understanding the AMF diversity within a specific region is crucial for maximizing their impact and potential applications. While previous studies have focused on individual AMF species, limited research has examined the overall impact of AMF communities on grapevine growth (Moukarzel et al. 2022). Previous research identified distinct AMF communities associated with different grapevine rootstocks, highlighting variations in AMF diversity across vineyard sites (Moukarzel et al. 2021; Noceto et al. 2024; Lailheugue et al. 2024a, b). Building upon previous findings, the main objective of this study was to investigate the functional effects of AMF communities on grapevine growth parameters and nutrient uptake by inoculating two commercial rootstocks (5 C and Schwarzmann) with AMF communities sourced from other rootstocks present at the same site. These rootstocks were selected based on their known influence on AMF community composition as reported by Moukarzel et al. (2021). In this study, we hypothesise that rootstocks will show increased growth and nutrient uptake when associated with their ‘home’ AMF communities, relative to inoculation with AMF communities originating from other rootstocks (‘away’ communities).

## Materials and methods

### Sample collection and AMF community recovery

Root samples from eight different rootstocks were collected from Muddy Water vineyard (43° 2′ 43.16″ S, 172° 47′ 17.23″ E) located in Waipara, North Canterbury (New Zealand). The trial in the vineyard was fully replicated with an 8 × 8 Latin Square design with eight rootstocks grafted onto Pinot Noir AM 10/5 (clone MS05001). All rootstocks were sourced from the same nursery. The roots were used to set-up trap cultures for AMF recovery as described in Moukarzel et al. (2021). AMF spores were isolated from trap cultures following the method of Daniels and Skipper (1982) and identified based on morphology and Sanger sequencing as outlined in Moukarzel et al. (2021). The AMF community recovered from the roots of each of the rootstocks is presented in Table 1.

**Table 1:** Arbuscular mycorrhizal fungal communities recovered from trap cultures associated with each grapevine rootstock. Spores were identified to genus level using Sanger sequencing (Moukarzel et al. 2021)

Rootstocks	Parentage	AMF spores recovered from trap cultures^1^ (‘home’ AMF community)
101 − 14	*V. riparia* × *V. rupestris*	Dominated by *Funneliformis* sp., followed by *Ambispora* sp., *Glomus* sp.1, and *Glomus* sp.2
5 C	*V. berlandieri* × *V. riparia*	Dominated by *Ambispora* sp. followed by *Funneliformis* sp., *Glomus* sp.1, and *Glomus* sp.2
Schwarzmann	*V. riparia* × *V. rupestris*	Dominated by *Funneliformis* sp., followed by *Ambispora* sp., *Glomus* sp.1, *Claroideoglomus* sp. and *Glomus* sp.3
Riparia Gloire	*V. riparia*	Dominated by *Ambispora* sp., followed by *Funneliformis* sp., *Glomus* sp.1, *Claroideoglomus* sp. and *Glomus* sp.2
420 A	*V. berlandieri* × *V. riparia*	Dominated by *Funneliformis* sp., followed by *Ambispora* sp., and *Glomus* sp.2
3309 C	*V. riparia* × *V. rupestris*	Dominated by *Funneliformis* sp., followed by *Ambispora* sp., *Glomus* sp.1, and *Glomus* sp.2
99R	*V. berlandieri* × *V. rupestris*	Dominated by *Funneliformis* sp., followed by *Ambispora* sp., *Glomus* sp.1, and *Glomus* sp.3
Fercal	*V. berlandieri* × Colombard (*V. vinifera*)	Dominated by *Funneliformis* sp., followed by *Glomus* sp.1 and *Glomus* sp.3

^**1**^ These communities will be considered as the “home” AMF communities for these rootstocks

### Experimental set-up

Dormant cuttings (one-year-old) of the selected grapevine rootstock cultivars (5 C and Schwarzmann) were acquired from Riversun Nursery in Gisborne, New Zealand. The grapevine cuttings were rooted in sterilized pumice using a mist propagation-bottom heat system maintained at 25 °C in the nursery at Lincoln University. The choice of rootstock cultivars (5 C and Schwarzmann) for this investigation was based on the distinct AMF communities obtained from trap cultures, as summarized in Table 1. To assess the influence of AMF communities, a controlled experiment employing a ‘home’ and ‘away’ approach was designed. In this experiment, the two rootstock cultivars were inoculated with their own ‘home’ AMF community and the ‘away’ AMF communities associated with the seven other rootstocks from the pot cultures, as outlined in Table 2.

**Table 2:** ‘Home’ and ‘away’ experimental layout for inoculation of Schwarzmann and 5 C rootstocks with AMF communities associated with different rootstocks from the Muddy Water vineyard

Rootstock	AMF Inoculum Source	Rootstock	AMF Inoculum Source	AMF Community
Schwarzmann	Schwarzmann	5 C	5 C	Home
Schwarzmann	5 C	5 C	Schwarzmann	Away 1
Schwarzmann	420 A	5 C	420 A	Away 2
Schwarzmann	99R	5 C	99R	Away 3
Schwarzmann	3309 C	5 C	3309 C	Away 4
Schwarzmann	101 − 14	5 C	101 − 14	Away 5
Schwarzmann	Fercal	5 C	Fercal	Away 6
Schwarzmann	Riparia Gloire	5 C	Riparia Gloire	Away 7

**Fig. 1:**
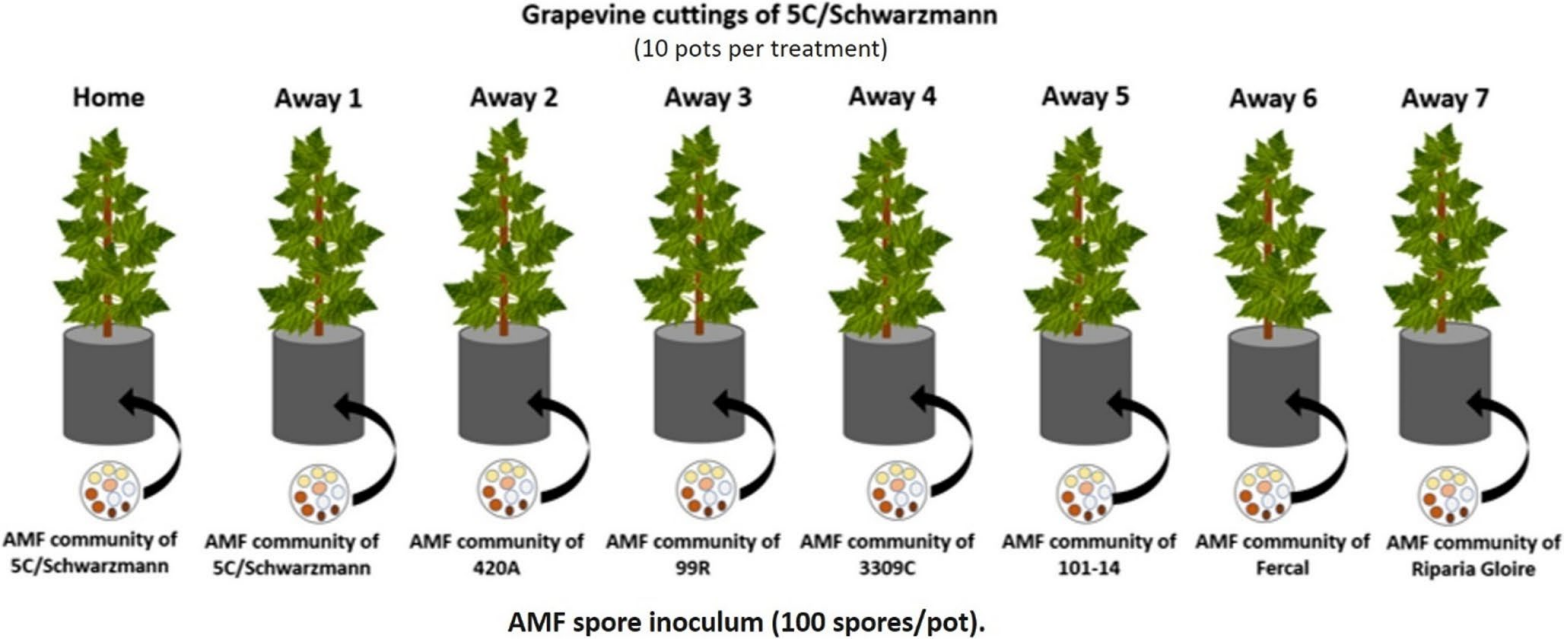
Schematic representation of the ‘home’ and ‘away’ experiment set-up. Grapevine cuttings from 5 C/Schwarzmann rootstocks planted into their own AMF communities (‘home’) and the ‘away’ AMF communities of 7 other rootstocks (Schwarzmann/5 C, 420 A, 99R, 3309 C, 101 − 14, Fercal, Riparia gloire)

The grapevine cuttings, which had been successfully rooted, were transplanted into 4 L pots filled with sterilised potting mix comprising 50% silica sand, 40% pumice, and 10% low-phosphorus potting mixture, including fertilizers: Osmocote 38-0-0 (3.0 g pot⁻¹), Osmocote 0-0-32 (1.5–2.0 g pot⁻¹), horticultural lime (1–2 g pot⁻¹), Micromax trace elements (1 g pot⁻¹), and Hydraflo (1 mL L⁻¹). These products were sourced from Everris International B.V., Geldermalsen, The Netherlands, and purchased from Intelligro Ltd., New Zealand. A hole was created within the growing media to accommodate the AMF spore inoculum obtained from pot cultures, following the methodology outlined in Moukarzel et al. (2021). Each pot received approximately 20 g of the AMF spore inoculum, containing an estimated count of 100 spores per 20 g (Supplementary Information, Table S1). To ensure direct contact between the grapevine rootstock and the inoculum, the rootstock was positioned at the center of the pot before being completely filled with the growing media (Fig. 1). The pots were arranged in a block design at the Lincoln University glasshouse, with ten replicates per treatment. As a control measure, uninoculated pots filled with the growing media but devoid of AMF inoculum were included in a random distribution among the blocks. These control pots were excluded from the subsequent analysis and solely served the purpose of identifying potential cross-contamination. Throughout the experiment, the grapevines were irrigated as needed, without the addition of any fertilizers. The plants were cultivated under natural lighting conditions for a duration of 5 months, during which the temperature inside the greenhouse ranged from 15 to 25 °C. All rootstocks were standardised to a single shoot for analysis. Any additional shoots or branching were removed to ensure consistency across treatments, and measurements were conducted on the primary stem only. The flowers or fruits produced were removed from the potted grapevine rootstocks.

### Grapevine growth parameters assessments

#### Shoot measurements

The shoot length (cm) of each grapevine plant was assessed at two and ten weeks after the initiation of the experiment to investigate the influence of AMF inoculation on shoot growth development during the early establishment of AMF symbiosis (two-week assessment) and later establishment (ten-week assessment). Shoot length was measured from the base of the shoot, at the point of attachment with the main rootstock cane, to the shoot tip using a tape measure.

#### Chlorophyll content measurements

In March of 2019, the chlorophyll content in the leaves of each grapevine plant was quantified using a SPAD 502 Plus (Konica Minolta, Inc., Tokyo, Japan) chlorophyll meter, following the methodology described in Moukarzel et al. (2022). For each plant, lower, middle, and upper leaves were selected for analysis. Three distinct measurements were obtained from different regions on the upper leaf surface, namely the base, middle, and tip of each leaf. Subsequently, the recorded SPAD measurements were utilized to determine the chlorophyll concentration in three replicates plants of each rootstock. The chlorophyll concentration was determined using the dimethylformamide (DMF) method described by Suzuki and Ishimaru (1990). For each rootstock (Schwarzmann and 5 C), a calibration curve was then produced using the SPAD measurements and the corresponding chlorophyll concentrations. The results were plotted in Excel and an R-squared value and line of best fit equation were generated (Supplementary Information, Figure S1).

#### Root and shoot biomass

In mid May (2019), the experiment was concluded, and the grapevine plants were harvested. The shoots were detached from the stem, carefully placed in labeled paper bags, and dried in an oven at 60 °C for 48 h. After the drying process, the shoots were weighed to determine their dry weight. The roots of each plant were washed to remove any adhering potting mix particles and subsequently cut at the base of the stem. Similar to the shoots, the roots were then placed in labeled paper bags and dried at 60 °C for 48 h. Following the drying period, the roots were weighed to determine their dry weight. This allowed for the determination of both shoot and root biomass for each individual plant.

#### AMF colonisation confirmation

At harvest, root samples (approximately 0.2 g) from three randomly selected plants per treatment were cut using scissors and transferred into 15 mL tubes. The roots were stored in a fridge until subsequently being stained with trypan blue in lactic acid following the methodology outlined in Moukarzel et al. (2020). Colonisation was assessed by microscopy to confirm the presence of AMF structures such as arbuscles, hyphae and vesicles.

#### Leaf macro- and micronutrients

Dried leaf samples (0.2 g) from the same 3 replicate plants used for determining AMF colonisation of roots for each treatment were ground to a fine powder and subjected to microwave-assisted acid digestion in in Teflon PFA^®^ vessels with Kevlar shielding using a CEM MARS Xpress system (CEM Corporation, North Carolina, USA), following the method described by Moukarzel et al. (2022). Following digestion, concentrations of micro- (B, Cu, Fe, Mn, and Zn) and macro-nutrients (Ca, K, Mg, P, and S) were determined using the pre-calibrated system. Results were expressed in ppm.

#### Data analysis

The effects of AMF treatment (i.e., ‘home’ and ‘away’) and rootstock and their interaction (rootstock × AMF) on grapevine growth parameters, nutrient uptake and chlorophyll content were tested using linear mixed-effects models. Generalised linear mixed-effects models (GLMMs) were fitted using the lme4 package in R (Bates et al. 2015). All analyses were conducted in R version 3.2.3 (R Core Team, 2019). Estimated marginal means and pairwise post hoc comparisons were calculated using the emmeans package (Lenth 2020). Type II/III analysis of variance tables and associated chi-square statistics and p-values were obtained using the Anova function implemented in the car package (Fox and Weisberg 2019). These response variables were modelled as a function of the AMF treatment (i.e., ‘home’ or ‘away’), considering the rootstock and AMF replicate as random factors. Total biomass of plants was modelled using a normal error distribution and shoot and root weight with a Gamma distribution (Zuur et al. 2009). The indirect influence of AMF on chlorophyll content was tested using the Pearson Correlation test of chlorophyll and macro- and micro- nutrient concentrations. The results showing significant differences/interactions (*p* < 0.05) were plotted in R or SigmaPlot (14.0) and graphs were created. The detailed analysis of this study is presented in the Suplementary Information (Tables S2-S8).

## Results

### AMF colonisation confirmation in roots

AMF colonization was verified through the visual observation of vesicles, arbuscules, and hyphae structures in the sampled roots of both the ‘home’ and ‘away’ AMF-inoculated rootstock treatments. The uninoculated internal check plants were uncolonised (Supplementary information Figure S2).

### Shoot growth

**Fig. 2:**
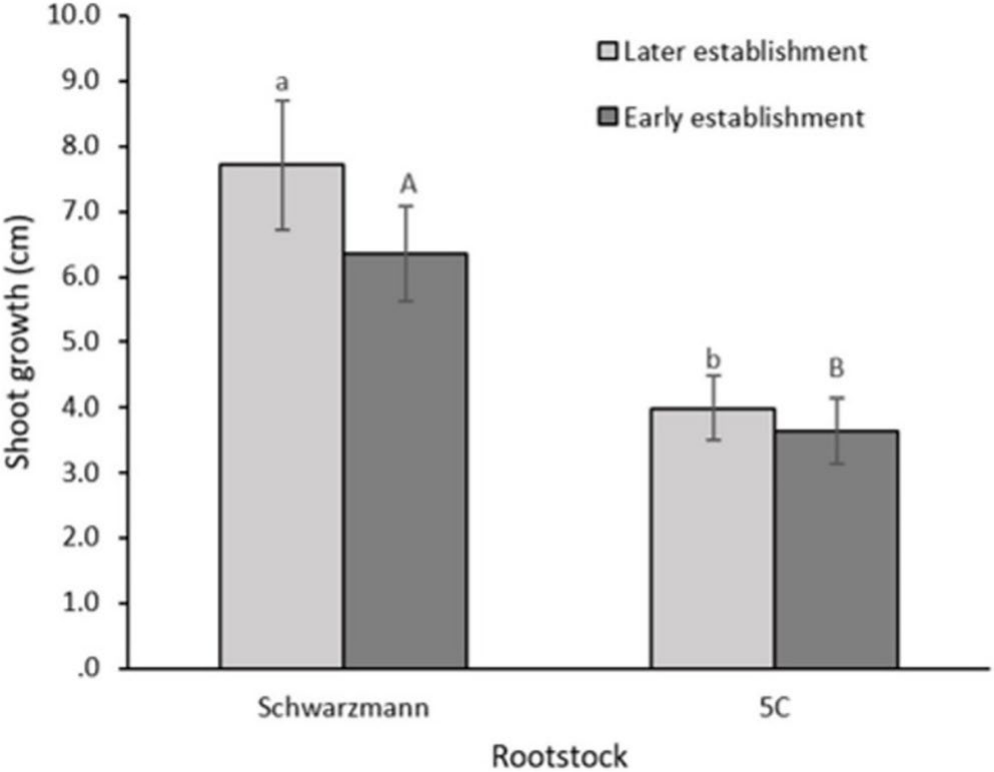
Effect of rootstocks on shoot growth during early (two weeks) and later (ten weeks) establishment. Bars with different letters are significantly different (*p* ≤ 0.05). Error bars show ±1SE

There was no significant main effect of AMF community establishment on shoot growth in either the early (two weeks, *p* = 0.636) or later (ten weeks, *p* = 0.235) periods (Supplementary Information, Table S2), but at the rootstock level, shoot growth significantly differed between rootstocks in both the early (*p* = 0.001) and later establishment (*p* = 0.003) periods. Shoot growth of Schwarzmann rootstock was significantly higher, by 50% and 40%, compared to that of 5 C rootstock in the early and later establishment periods, respectively (Fig. 2).

### Total chlorophyll content

**Fig. 3:**
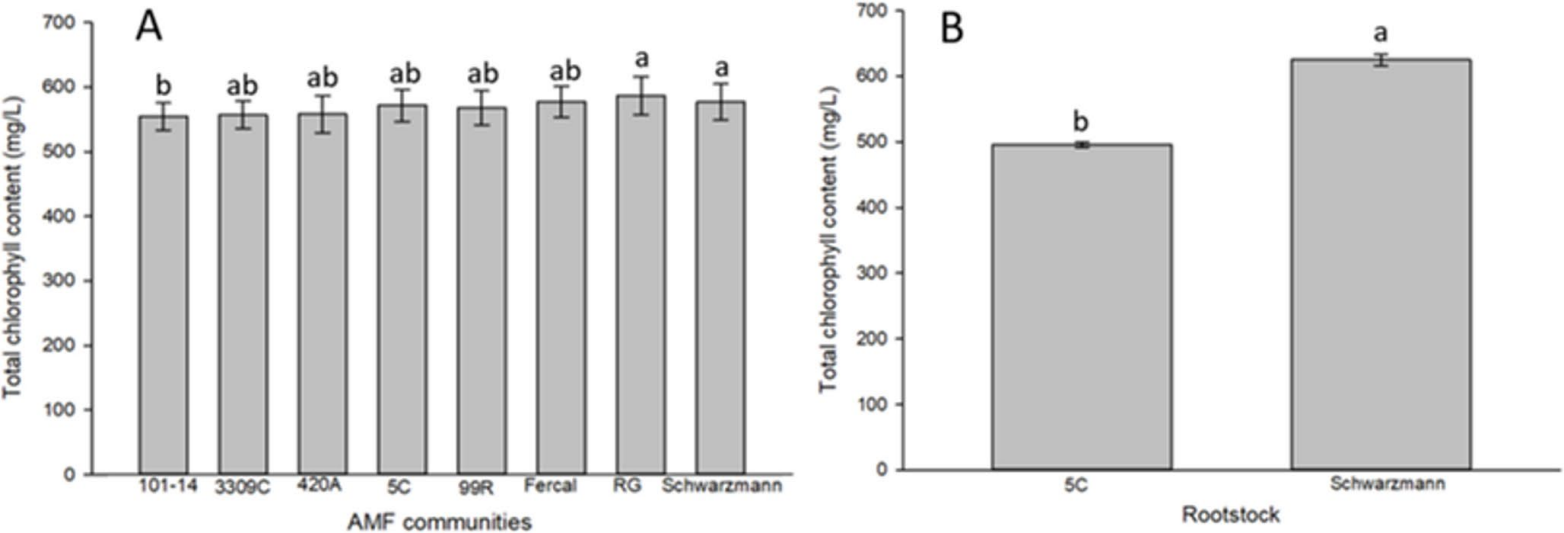
Effect of AMF communities (**A**) and rootstock (**B**) on total chlorophyll content in leaves where rootstocks (Schwarzmann and 5C) were inoculated with their own AMF communities and the AMF communities of other rootstocks. Bars with different letters are significantly different (*p* ≤ 0.05). Error bars show ±1SE. RG: Riparia Gloire

Separate standard curves were produced for each rootstock and the linear equation used to calculate the total chlorophyll content from the SPAD measurements were different for each rootstock (Supplementary Information, Figure S1). There was no significant effect of the ‘home’ and ‘away’ treatments on total chlorophyll content (*p* = 0.732). However, both the AMF community and the rootstock had a significant effect (*p* = 0.002 and *p* < 0.001, respectively) on the total chlorophyll content. Rootstocks inoculated with AMF communities from Schwarzmann and Riparia Gloire had 5% and 8% higher chlorophyll content than rootstocks inoculated with AMF communities from 101 − 14 (Fig. 3, A). There was no significant difference in the chlorophyll content of rootstocks inoculated with the other AMF communities compared with rootstocks inoculated with AMF communities from 101 − 14, Riparia Gloire or Schwarzmann. The chlorophyll content in Schwarzmann leaves was 26% higher compared to 5 C rootstock (Fig. 3, B). Moreover, there was no interaction between the rootstock and the AMF community (*p* > 0.05). The detailed summary of the statistical analysis for total chlorophyll content in grapevine leaf samples with the effects of ‘home’ and ‘away’ treatment, AMF community, rootstock and their interaction are presented in Supplementary Information, Table S3.

### Biomass analysis at harvest

**Fig. 4:**
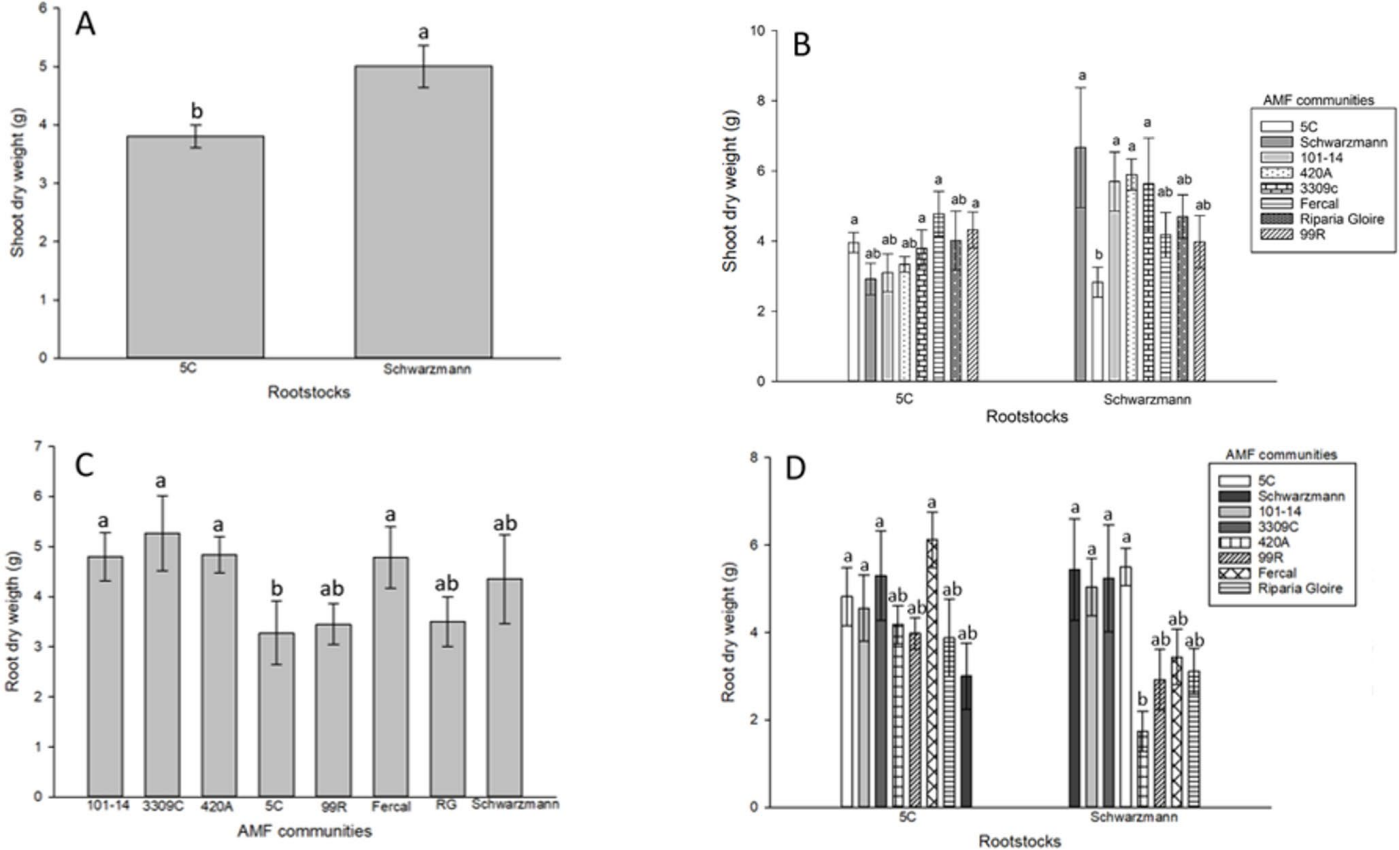
Effect of AMF communities, rootstocks, and their interaction on shoot dry weight (**A** & **B**), and root dry weight (**C**), where rootstocks (5 C and Schwarzmann) were inoculated with their own AMF communities and the AMF communities of other rootstocks. Bars with different letters are significantly different (*p* ≤ 0.05). Error bars show ±1SE

The influence of rootstock, AMF community, and their interaction on these parameters were examined (Supplementary information, Table S4). Shoot biomass significantly differed among rootstocks (*p* = 0.007). The shoot dry weight of Schwarzmann was 25% higher than that of 5 C rootstock (Fig. 4, A). Moreover, there was a significant interaction between AMF community and rootstock on shoot dry weight (*p* < 0.001, Fig. 4, B). Schwarzmann vines inoculated with AMF community from 5 C had significantly lower shoot dry weight compared to Schwarzmann vines inoculated with AMF communities from 3309 C (by 62%), 101 − 14 (by 65%), 420 A (by 70%) and Schwarzmann (by 75%). AMF community had no significant effect on the shoot dry weight of 5 C.

The ‘home’ and ‘away’ treatments had a significant effect on plant biomass (*p* < 0.05). Root and shoot dry weight were 15% and 20% higher respectively, in rootstocks inoculated with their ‘home’ AMF communities compared to the ‘away’ communities (Fig. 4, B & D).

Root biomass significantly differed among AMF communities (*p* < 0.001). Rootstocks inoculated with AMF community from 5 C had 25% lower root dry weight compared with rootstocks inoculated with AMF communities from 101 − 14, 420 A and Fercal, and 30% lower root dry weight compared with rootstocks inoculated with AMF communities from 3309 C respectively (Fig. 4, C). There was a significant interaction (*p* < 0.001) between AMF community and rootstocks on root dry weight (Fig. 4, D). Schwarzmann vines inoculated with AMF community from 420 A had significantly lower root dry weight compared to Schwarzmann vines inoculated with AMF communities from 5 C (by 241%), 3309 C (by 223%), 101 − 14 (by 205%), and Schwarzmann (by 235%). No significant difference was observed with 5 C.

### Macro- and micro-nutrients uptake

The ‘home’ and ‘away’ treatment had no significant effect on macro-nutrients (Ca, K, Mg, P & S) or micronutrients in leaves (B, Cu, Fe, Mn & Zn) (*p* > 0.05). There were a significant effect of rootstock and the AMF community on B and Cu. Boron levels were significantly (*p* = 0.010) affected by the rootstock, with the level in the leaves of Schwarzmann vines (18.01 ppm) being significantly higher than in those of 5 C vines (14.52 ppm). Copper levels were significantly influenced by the interaction effects of AMF communities and rootstock (*p* = 0.013). The level in the leaves of Schwarzmann vines grown in 5 C AMF community (3.60 ppm) and the level in the leaves of Schwarzmann vines grown in Fercal AMF community (3.69 ppm) being significantly higher than in the leaves of Schwarzmann vines grown with 101 − 14 (2.39 ppm), 3309 C (2.20 ppm) and 420 A (2.02 ppm) AMF community. See Supplementary information, Tables S5-S7 for the full statistical analysis for macro- and micro- nutrient in grapevine leaf samples.

The Pearson correlation test was also performed to study the correlation between total chlorophyll content and the macro- and micro- nutrient concentration in grapevine leaf samples. Chlorophyll has a significant positive correlation with Mn (*p* = 0.006) and Zn (*p* = 0.050) and a significant negative correlation with B (*p* = 0.008) (Supplementary information, Table S8).

## Discussion

This study aimed to address a critical knowledge gap concerning the functional role of AMF communities associated with different grapevine rootstocks. By comparing the effects of ‘home’ and ‘away’ AMF communities, originally isolated from distinct rootstocks, we evaluated whether rootstock-specific AMF assemblages influence grapevine growth and nutrient acquisition. This approach provides insights into the extent to which intra-genus host identity shapes AMF functionality and plant response under controlled conditions. This differs from reductionist studies where the beneficial effects of only one or two AMF species are investigated (Lumini et al. 2010; Cangahuala-Inocente et al. 2011; Holland et al. 2013; Nogales et al. 2019) and instead treats the AMF community as a functional unit. The positive effect of mycorrhizal fungi on plant growth parameters is dependent on community diversity and composition, and studies have shown that a mixture of AMF species is more effective than a monospecific inoculum as AMF functions are inter-related (Jansa et al. 2008; Sharma et al. 2009; Gogoi and Singh 2011). Moreover, by using the whole AMF communities identified for each rootstock, the data presented here is more realistic to what is happening in the vineyards (Moukarzel et al. 2021; Noceto et al. 2024).

This work shows that the degree of positive effect gained from AMF occurs at the community-level. It supports previous research indicating the responsiveness of grapevines to mycorrhiza depends on the composition and origin of the AMF community, as well as the host plant’s genotype and environmental context (*review* by Trouvelot et al. 2015). The specific effects of inoculation varied depending on the AMF communities, rootstocks, and their interactions. Similar observations were made in prior studies by Ozdemir et al. (2010) and Bleach et al. (2008), where the growth response of different rootstocks depended on the AMF communities and their interactions.

This study demonstrates the benefits of specific AMF communities to grapevine vigour. Root and shoot biomass were improved when grown in ‘home’ compared to ‘away’ AMF communities for Schwarzmann rootstocks. These findings indicate that the performance of these rootstocks is partially influenced by their ability to recruit compatible AMF communities. Klironomos (2003) demonstrated that AMF isolated from the same plant species conferred greater growth benefits than AMF originating from unrelated hosts, particularly when comparing across distinct plant genera. While our study involves only *Vitis* rootstocks, all within the same genus and commonly used in viticulture, they are genetically distinct and derived from different wild species such as *V. riparia*, *V. rupestris*, and *V. berlandieri*. This narrower phylogenetic range may explain the absence of strong host-specific AMF effects observed in our experiment. Incorporating rootstock parentage into AMF studies can help clarify how intra-genus host relatedness influences AMF recruitment and function.

The findings of this study highlight variation in the responses of rootstocks to the AMF communities impacting plant growth parameters. An increase in shoot and root dry weights was seen for Schwarzmann when planted into the AMF communities of some of the other rootstocks compared to 5 C where plant growth was lower. This could indicate that Schwarzmann is more responsive to colonisation by a wide diversity of AMF compared with 5 C. This was observed in the original pot cultures where the Schwarzmann AMF community was more diverse (*Funneliformis* sp., *Ambispora* sp., *Glomus* spp. and *Claroideoglomus* sp.) and the spore abundance of each species was higher compared with the AMF community associated with 5 C (*Funneliformis* sp., *Ambispora* sp. and *Glomus* spp.) which was less diverse and abundant. This was also reported in other studies, where it was shown that the outcome of the AM symbiosis could differ according to the genotype of the two symbionts, host plants, and AMF, as well as their combination (Lee et al. 2013; Chandrasekaran, 2014). Studies have indicated that Glomeraceae family members, including *F. mosseae*, *Rhizophagus irregularis*, and *G. aggregatum*, exhibit higher intraradical colonization rates compared to other families (Hart and Reader 2005). These species serve as a vital functional group, promoting plant growth and enhancing nutrition (Engelmoer et al. 2014; Giovannini et al. 2020).

Contrary to the expectation, AMF did not stimulate grapevine shoot growth in the early or later establishment of the symbiosis. Shoot growth was however different between the rootstocks. Schwarzmann had a higher shoot growth compared to 5 C in the early establishment stage. According to Goldammer (2018), 5 C is a moderate to vigorous rootstock, with Schwarzmann having a low to moderate vigour. However, in this study it may be that Schwarzmann has an initial fast growth rate compared to 5 C.

Nutrient analysis revealed differences in grapevine leaf uptake of key elements particularly copper (Cu), and boron (B)—that varied depending on both the AMF community and the rootstock genotype under low-P soil conditions. These responses were not consistent across rootstocks, indicating that the effects of AMF communities on nutrient uptake were rootstock-specific rather than simply driven by community origin. In some cases, differences were only observed for Schwarzmann, while 5 C showed limited responsiveness to AMF community variation. These findings suggest that the composition of the AMF community can influence nutrient uptake efficiency, but that this effect is strongly dependent on host genotype.

In plant growth and development, chlorophyll contributes to energy production through photosynthesis (Alam et al. 2019). In the current study, chlorophyll content in grapevine leaves were increased by the AMF communities of Schwarzmann and of Riparia Gloire in respect to 101 − 14 AMF community. This could be due to the presence of specific AMF species in the inoculum which caused the increase in chlorophyll content (also suggested in Moukarzel et al. 2022). AMF communities associated with Schwarzmann and Riparia Gloire were relatively diverse and dominated by members of *Glomus* spp. and *Funneliformis* sp., which may have contributed to the observed increases in chlorophyll content. Previous work has shown that grapevines inoculated with AMF consortia, including taxa from these genera, can exhibit enhanced chlorophyll levels (Ye et al. 2022). Our results extend these findings by suggesting that even AMF communities originating from the same vineyard site, but associated with different rootstocks, can differentially influence plant physiological traits. The response of chlorophyll content did not consistently align with a ‘home’ versus ‘away’ pattern, indicating that AMF effects may be trait-specific and influenced by functional differences among taxa or their relative abundance, rather than by community origin alone. It is therefore possible that AMF communities associated with Schwarzmann and Riparia Gloire contained taxa or dominance patterns that were particularly effective at influencing chlorophyll accumulation, even in rootstocks that were otherwise less responsive to AMF. Chlorophyll analysis further revealed differences between rootstocks, with Schwarzmann exhibiting higher total chlorophyll levels than 5 C. This variation is likely attributable to inherent differences in rootstock genotype, physiology, root system architecture, and vigour. Similar variation in chlorophyll content among grapevine rootstocks has been reported in previous studies (Koblet et al. 1996; Bica et al. 2000; Keller et al. 2001; Gargin, 2011; Ulas et al. 2013; Somkuwar et al. 2015; Blank et al. 2018).

In conclusion, the study highlights the importance of AMF communities in influencing rootstock growth in a rootstock-specific manner. It also suggests complementarity within AMF communities, where some AMF species have beneficial effects on above or below ground biomass while others increase nutrient uptake which indirectly affects the chlorophyll content in plants. Further studies should examine how AMF influence the production of key metabolites, such as phenolic compounds, which contribute to berry composition and ultimately wine quality (Torres et al. 2016, 2018). Importantly, the AMF communities used in this study were originally associated with rootstocks grafted to Pinot noir, and this could influence the recruitment of AMF communities and their functional effects (Darriaut et al. 2022; Lailheugue et al., 2024a, 2024b). Future investigations into these interactions may provide a clear understanding of the AMF role in potentially modulating nutrient uptake and metabolite profiles in grapevines.

## Supplementary Information

Below is the link to the electronic supplementary material.

Supplementary Material 1 (DOCX 771 KB)

## Data Availability

The original contributions presented in the study are included in the article. Further inquiries can be directed to the corresponding authors.
