# Supplementary material for: Root-derived AMF communities modulate growth and nutrient dynamics in grapevine rootstocks
Source: Mycorrhiza. 2026 May 7;36(3):20. doi: 10.1007/s00572-026-01264-5 (PMC13152886; doi:10.1007/s00572-026-01264-5)
Supplement: Supplementary file 1 — Supplementary Material 1 (DOCX 771 KB) [file 572_2026_1264_MOESM1_ESM.docx]

1. Supplementary information

Table S1. Spore count/abundance in 20 g of medium culture

| Number of Spores/20 g | |
| --- | --- |
| XXXX High | >40 |
| XXX Moderately High | 30-40 |
| XX Low | 20-30 |
| X Very Low | 10 to 20 |

| **Rootstocks** | ***Funneliformis* sp.** | ***Ambispora* sp.** | **Glomus sp.1** | **Claroideoglomus sp.** | **Glomus sp.3** |
| --- | --- | --- | --- | --- | --- |
| Schwarzmann | XXXX | XXX | XX | X | X |
| Riparia Gloire | XXXX | XXX | XX | XX | X |
| 101-14 | XXXX | X | XX |  | XXX |
| 5C | XXX | XXXX | XX |  | X |
| 3309C | XXXX | XXX | XX |  | X |
| 420A | XXXX | XXX |  | X | XX |
| Fercal | XXX |  | XX |  | X |
| 99R | XXXX | XXX | XX |  | X |

Chlorphyll calibration curves for total chlorophyll measurements

For each rootstock a calibration curve was produced using the SPAD measurements and the corresponding chlorophyll concentrations. An R-squared value and line of best fit equation were generated.

**Figure S1.** Calibration curve for SPAD measurements and the corresponding chlorophyll concentrations in leaves of the three grapevine rootstocks.


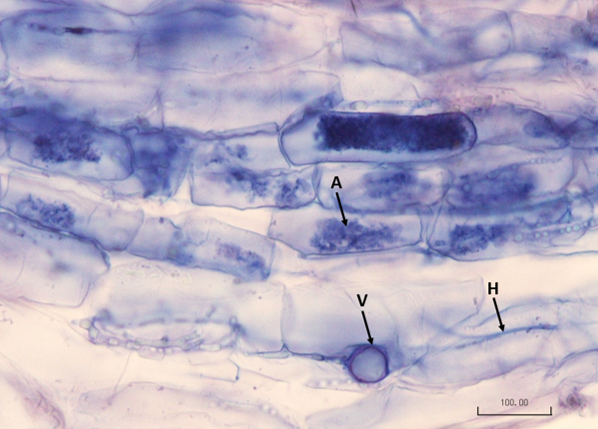

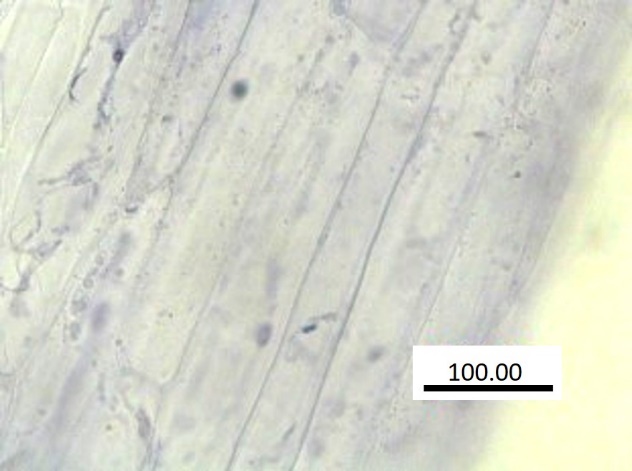


**B**

**A**

**Figure S2.** Representative micrograph of confirmation of AMF colonisation of Trypan blue stained grapevine roots. A) AMF inoculated, B) uninoculated internal checks. V: vesicle, A: arbuscules, H: hyphae.

Table S2. Statistical significance of the fixed effects for the shoot length analysis for the early (two weeks) and later (10 weeks) establishment assessments. Df- degrees of freedom.

| Time | Fixed effects | Sum of squares | Df | Mean Square | F | Sig. |
| --- | --- | --- | --- | --- | --- | --- |
| Later establishment | Rootstock | 555.025 | 1 | 555.025 | 11.531 | 0.001*** |
| Early establishment | Rootstock | 294.306 | 1 | 294.306 | 9.370 | 0.003** |
| Later establishment | AMF community | 269.800 | 7 | 38.543 | 0.742 | 0.636 |
| Early establishment | AMF community | 305.844 | 7 | 43.692 | 1.341 | 0.235 |

*= significant at p<0.01, ** = significant at p<0.001, *** = significant at p<0.0001.

Table S3. Statistical significance of the fixed effects for the total chlorophyll content. Df- degrees of freedom.

| **Response** | **Fixed effects** | **Chi squares** | **Df** | ***p* values** |
| --- | --- | --- | --- | --- |
| Chlorophyll content | Treatment | 0.1167 | 1 | 0.733 |
|  | AMF communities | 21.8361 | 7 | 0.003*** |
|  | Rootstock | 201.3884 | 1 | <0.001*** |
|  | AMF × Rootstocks | 3.7785 | 6 | 0.707 |

*= significant at p<0.01, ** = significant at p<0.001, *** = significant at p<0.0001.

Table S4. Statistical significance of the fixed effects for the shoot and root dry weight. Df- degrees of freedom.

| **Fixed effects** | **Chi squared** | | **Df** | **P value** |
| --- | --- | --- | --- | --- |
|  | **Shoot dry weight** |  | |  |
| Treatment | 5.8538 | 1 | | 0.015* |
| AMF communities | 7.9936 | 7 | | 0.333 |
| Rootstock | 7.2576 | 1 | | 0.007** |
| AMF x Rootstocks | 25.6415 | 7 | | 0.001*** |
|  | **Root dry weight** |  | |  |
| Treatment | 11.679 | 1 | | <0.001*** |
| AMF communities | 37.7145 | 7 | | <0.001*** |
| Rootstock | 4.1688 | 1 | | 0.051 |
| AMF x Rootstocks | 24.7076 | 7 | | 0.001*** |

*= significant at p<0.01, ** = significant at p<0.001, *** = significant at p<0.0001.

Table S5. Statistical significance of the fixed effects on the macro- and micro-nutrients. Df- degrees of freedom.

| **Response** | **Fixed effects** | **Chi squares** | **Df** | ***p* values** |
| --- | --- | --- | --- | --- |
| B | Treatment | 0.1329 | 1 | 0.715 |
|  | Rootstock | 9.0594 | 2 | 0.010* |
|  | AMF communities | 9.7461 | 7 | 0.203 |
|  | AMF x Rootstocks | 14.6022 | 9 | 0.102 |
| Ca | Treatment | 0.5164 | 1 | 0.472 |
|  | AMF communities | 7.0239 | 7 | 0.426 |
|  | Rootstock | 2.2104 | 2 | 0.331 |
|  | AMF x Rootstocks | 8.3962 | 9 | 0.494 |
| Cu | Treatment | 1.288 | 1 | 0.256 |
|  | AMF communities | 3.3109 | 7 | 0.854 |
|  | Rootstock | 0.1539 | 2 | 0.925 |
|  | AMF x Rootstocks | 20.8831 | 9 | 0.013* |
| Fe | Treatment | 0.0997 | 1 | 0.752 |
|  | AMF communities | 11.9913 | 7 | 0.100 |
|  | Rootstock | 1.4702 | 2 | 0.479 |
|  | AMF x Rootstocks | 5.3180 | 9 | 0.805 |
| K | Treatment | 0.3368 | 1 | 0.561 |
|  | AMF communities | 9.0004 | 7 | 0.252 |
|  | Rootstock | 1.8116 | 2 | 0.404 |
|  | AMF x Rootstocks | 11.4621 | 9 | 0.245 |
| Mg | Treatment | 0.2545 | 1 | 0.613 |
|  | AMF communities | 5.8371 | 7 | 0.558 |
|  | Rootstock | 1.7357 | 2 | 0.419 |
|  | AMF x Rootstocks | 10.6238 | 9 | 0.302 |
| P | Treatment | 0.0241 | 1 | 0.876 |
|  | AMF communities | 7.5411 | 7 | 0.374 |
|  | Rootstock | 0.0843 | 2 | 0.958 |
|  | AMF x Rootstocks | 3.2793 | 9 | 0.952 |
| Zn | Treatment | 0.3164 | 1 | 0.573 |
|  | AMF communities | 4.6221 | 7 | 0.705 |
|  | Rootstock | 0.8431 | 2 | 0.656 |
|  | AMF x Rootstocks | 15.3145 | 9 | 0.082 |

*= significant at p<0.01, ** = significant at p<0.001, *** = significant at p<0.0001.

Table S6. The mean concentration (ppm) of different nutrients in the leaves of different rootstocks. Statistical significance of the rootstock effect for the nutrient analysis. Different letters mean they are significantly different (p ≤ 0.05). Mean ±1SE.

|  | **Nutrient concentration (ppm) in rootstock leaves** | |  |
| --- | --- | --- | --- |
| **Nutrients** | **5C** | **Schwarzmann** | **p value** |
| **B** | 14.523 ± 0.35 **b** | 18.01 ± 1.205 **a** | 0.010* |
| **Cu** | 2.74 ± 0.186 **a** | 2.73 ± 0.132 **a** | 0.925 |
| **Fe** | 65.99 ± 2.81 **a** | 73.39 ± 2.57 **a** | 0.479 |
| **K** | 17066.40 ± 1013.73 **a** | 14011.48 ± 1436.38 **a** | 0.404 |
| **Mg** | 6241.85 ± 293.33 **a** | 6369.79 ± 195.91 **a** | 0.418 |
| **Mn** | 232.08 ± 12.88 **a** | 181.86 ± 8.58 **a** | 0.326 |
| **P** | 964.94 ± 84.81 **a** | 992.56 ± 63.09 **a** | 0.958 |
| **S** | 1743.85 ± 84.72 **a** | 1773.59 ± 41.35 **a** | 0.521 |
| **Zn** | 23.52 ± 0.89 **a** | 20.63 ± 0.92 **a** | 0.656 |

*= significant at p<0.01, ** = significant at p<0.001, *** = significant at p<0.0001.

Table S7. The mean concentration (ppm) of different nutrients in the leaves of the different rootstocks grown in the different AMF communities. Statistical significance of the AMF community and rootstock interaction effect for the nutrient analysis. Different letters mean they are significantly different (p ≤ 0.05). Mean ±1SE.

|  |  | **Nutrient concentration (ppm) in rootstock leaves** | | | | | | | | |
| --- | --- | --- | --- | --- | --- | --- | --- | --- | --- | --- |
| AMF community | Rootstock | B | Cu | Fe | K | Mg | Mn | P | S | Zn |
| 101-14 | 5C | 28.07 ± 7.80 **a** | 3.30 ± 0.11 **ab** | 63.12 ± 10.50 **a** | 24612.83 ± 9277.95 **a** | 5549.440 ± 535.27 **a** | 211.47 ± 37.61 **a** | 1051.10 ± 365.75 **a** | 1753.82 ± 87.44 **a** | 23.42 ± 0.69 **a** |
| 3309C | 5C | 16.96 ± 2.07 **a** | 2.73 ± 0.34 **ab** | 78.25 ± 10 **a** | 14211.59 ± 2593.29 **a** | 6189.587 ± 256.91 **a** | 220.82 ± 12.69 **a** | 1008.99 ± 79.44 **a** | 1686.16 ± 100.48 **a** | 18.30 ± 2.13 **a** |
| 420A | 5C | 16.16 ± 1.22 **a** | 2.93 ± 0.07 **ab** | 82.53 ± 6.21 **a** | 9069.66 ± 899.06 **a** | 6774.013 ± 469.25 **a** | 141.28± 17.96 **a** | 838.27 ± 69.89 **a** | 1776.96 ± 32.19 **a** | 20.11 ± 0.48 **a** |
| 5C | 5C | 16.78 ± 0.73 **a** | 2.35 ± 0.24 **ab** | 70.14 ± 4.56 **a** | 15125.06 ± 2811.01 **a** | 5968.387 ± 505.37 **a** | 184.94 ± 35.17 **a** | 1102.56 ± 240.07 **a** | 1785.41 ± 76.25 **a** | 19.24 ± 1.69 **a** |
| 99R | 5C | 16.36 ± 1.21 **a** | 2.27 ± 0.21 **ab** | 82.14 ± 5.38 **a** | 11565.80 ± 1136.52 **a** | 6116.803 ± 644.19 **a** | 162.88 ± 11.64 **a** | 985.39 ± 170.76 **a** | 1730.33 ± 102.69 **a** | 18.11 ± 0.57 **a** |
| Fercal | 5C | 16.45 ± 1.34 **a** | 2.29 ± 0.11 **ab** | 79.95 ± 5.72 **a** | 12508.07 ± 1507.60 **a** | 6369.697 ± 332.70 **a** | 202.13 ± 17.54 **a** | 841.76 ± 95.97 **a** | 1701 ± 83.10 **a** | 18.68 ± 0.26 **a** |
| Riparia Gloire | 5C | 18.16 ± 1.32 **a** | 3.13 ± 0.77 **ab** | 60.79 ± 1.92 **a** | 12840.49 ± 1964.14 **a** | 6986.730 ± 877.16 **a** | 172.72 ± 14.16 **a** | 1160.32 ± 182.21 **a** | 1875.41 ± 274.94 **a** | 24.88 ± 5.40 **a** |
| Schwarzmann | 5C | 15.16 ± 1.06 **a** | 2.82 ± 0.46 **ab** | 70.17 ± 4.12 **a** | 12158.38 ± 2304.09 **a** | 7003.697 ± 685.42 **a** | 158.68 ± 16.71 **a** | 951.82 ± 214.52 **a** | 1879.70 ± 145.95 **a** | 22.32 ± 4.2 **a** |
| 101-14 | Schwarzmann | 13.97 ± 1.64 **a** | 2.39 ± 0.18 **b** | 63.51 ± 4.95 **a** | 15964.70 ± 1034 **a** | 5456.140 ± 876.95 **a** | 220.67 ± 11.53 **a** | 727.74 ± 83.52 **a** | 1620.44 ± 100.49 **a** | 20.73 ± 1.09 **a** |
| 3309C | Schwarzmann | 14.92 ± 0.32 **a** | 2.2 ± 0.23 **b** | 58.63 ± 6.84 **a** | 17209.13 ± 4122.43 **a** | 6097.677 ± 740.35 **a** | 326.16 ± 40.05 **a** | 895.88 ± 79.39 **a** | 1635.52 ± 151.55 | 23.29 ± 1.34 **a** |
| 420A | Schwarzmann | 12.80 ± 0.83 **a** | 2.02 ± 0.23 **b** | 59 ± 10.77 **a** | 16098.60 ± 1729.08 **a** | 5438.327 ± 254.28 **a** | 209.32 ± 29.78 **a** | 688.85 ± 72.15 **a** | 1518.25 ± 46.04 **a** | 20.05 ± 2.85 **a** |
| 5C | Schwarzmann | 15.09 ± 0.62 **a** | 3.60 ± 0.63 **a** | 68.19 ± 3.87 **a** | 19549.90 ± 1565.92 **a** | 8053.453 ± 1600.83 **a** | 238.81 ± 24.19 **a** | 1424.94 ± 535.68 **a** | 2215.02 ± 442.54 **a** | 28.54 ± 3.75 **a** |
| 99R | Schwarzmann | 14.59 ± 0.79 **a** | 3.12 ± 0.51 **ab** | 76.09 ± 9.18 **a** | 23635.53 ± 3276.94 **a** | 6975.103 ± 634.62 **a** | 276.92 ± 15.84 **a** | 1219.73 ± 330.55 **a** | 2149.88 ± 301.94 **a** | 23.75 ± 0.36 **a** |
| Fercal | Schwarzmann | 15.72 ± 0.51 **a** | 3.69 ± 0.61 **a** | 79.58 ± 12.98 **a** | 18176.50 ± 2675.07 **a** | 6808.20 ± 247.44 **a** | 214.00 ± 24.54 **a** | 813.03 ± 92.11 **a** | 1686.47 ± 16.27 **a** | 26.84 ± 1.48 **a** |
| Riparia Gloire | Schwarzmann | 15.67 ± 0.58 **a** | 2.30 ± 0.43 **ab** | 57.12 ± 6.38 **a** | 11259 ± 2662.10 **a** | 6477.153 ± 44.13 **a** | 142.46 ± 5.305 **a** | 1148.93 ± 54.83 **a** | 1437.74 ± 111.42 **a** | 19.39 ± 1.34 **a** |
| Schwarzmann | Schwarzmann | 15.98 ± 2.60 **a** | 2.79 ± 0.96 **ab** | 67.63 ± 9.64 **a** | 14672.85 ± 3151.65 **a** | 5022.155 ± 1226.93 **a** | 269.64 ± 6.02 **a** | 109.48 ± 884.66 **a** | 1754.69 ± 330.39 **a** | 25.46 ± 4.77 **a** |
|  | p value | 0.10246 | 0.01318* | 0.8058 | 0.2454 | 0.3024 | 0.3642 | 0.9522 | 0.0987 | 0.08265 |

*= significant at p<0.01, ** = significant at p<0.001, *** = significant at p<0.0001.

**Table S8** Pearson correlation test of chlorophyll and macro- and micro- nutrient concentrations in grapevine leaf samples. Shaded cells highlight correlations which are significant at p ≤ 0.05.

|  |  | **Nutrients** | | | | | | | | |
| --- | --- | --- | --- | --- | --- | --- | --- | --- | --- | --- |
|  |  | **B** | **Cu** | **Fe** | **K** | **Mg** | **Mn** | **P** | **S** | **Zn** |
| **Chlorophyll** | Pearson Correlation | -0.378 | 0.056 | -0.195 | 0.172 | -0.026 | 0.394 | 0.025 | 0.010 | 0.284 |
|  | Sig. (2-tailed) | 0.008** | 0.707 | 0.184 | 0.244 | 0.859 | 0.006** | 0.867 | 0.949 | 0.050* |
|  | N | 48 | 48 | 48 | 48 | 48 | 48 | 48 | 48 | 48 |

*= significant at *p*=0.01; ** = significant at *p*=0.001, *** = significant at *p*=0.0001.
